# Attentional advantages in video-game experts are not related to perceptual tendencies

**DOI:** 10.1038/s41598-018-23819-z

**Published:** 2018-04-03

**Authors:** Nicole H. L. Wong, Dorita H. F. Chang

**Affiliations:** Department of Psychology, The University of Hong Kong, Hong Kong, Hong Kong

## Abstract

Previous studies have suggested that extensive action video gaming may enhance perceptual and attentional capacities. Here, we probed whether attentional differences between video-game experts and non-experts hold when attention is selectively directed at global or local structures. We measured performance on a modified attentional-blink task using hierarchically structured stimuli that consisted of global and local elements. Stimuli carried congruent or incongruent information. In two experiments, we asked observers to direct their attention globally (Experiment 1) or locally (Experiment 2). In each RSVP trial, observers were asked to identify the identity of an initial target (T1), and detect the presence or absence of a second target (T2). Experts showed a markedly attenuated attentional blink, as quantified by higher T2 detection sensitivity, relative to non-experts, in both global and local tasks. Notably, experts and non-experts were comparably affected by stimulus congruency. We speculate that the observed visuo-attentional advantage is unlikely to be related to mere differences perceptual tendencies (i.e., greater global precedence), which has been previously associated with diminished attentional blink.

## Introduction

Extensive video gaming has been traditionally associated with negative consequences such as poor physical health, low social motivation or even violence. In the past few decades, a considerable number of studies have suggested that video games, especially of the “action” variety, can enhance visual attention, cognitive abilities, perceptual skills, and even improve brain malleability^1,2^. Action video games generally refer to games that require fast responses and exhibition of high visuomotor coordination, while maintaining vigilant monitoring of the periphery for unexpected events and simultaneous tracking of multiple objects^1,3^. Different game genres account for their own specific skill improvements^3,4^. Improvements are typically found in observers who engage in action video-games extensively for a long period of time although the same enhancements can be seen in non-gamers who participate in video games for as few as ten hours, albeit to a lesser degree^2,5–9^. Despite these intriguing results, some have failed to find differences in perceptual (or more broadly cognitive) capacities between action video-gamers and non-video gamers^10,11^.

One line of studies seems to indicate that action video-gamers (AVGs) have larger visual attentional capacities, a broader visual field and higher visual acuity as compared to non-video-gamers (NVGs)^1,2,5^. For example, Green and Bavelier^5^ showed that task-irrelevant information presented in the form of flankers is processed to a greater extent by, and correspondingly affected performance on a target task more significantly for AVGs as compared to NVGs. Specifically, while flanker interference generally declined with increases in the difficulty of the target task in NVGs, AVGs continued to be affected even at higher difficulty levels. The authors attributed this difference in flanker interference between the two groups to reflect greater attentional resources in the expert group.

Increased attentional capacity in AVGs may also explain better performance in visuo-attentional tasks under transient-presentations. One oft-used paradigm involves rapid serial visual presentation (RSVP) and measures of an *attentional blink*. The attentional blink refers impaired processing of the second target within a set of two targets presented in close temporal succession^12,13^. That is, in a RSVP stream of items, participants consistently fail to report the identity or presence of the second target if identification of the first is required. This ‘blink’ is particularly pronounced for stimulus onset asynchronies (SOA) of 200 to 500 ms^12^. Accuracy generally recovers after 500 ms yet there are a few exceptions such as non-blinkers, who never or seldom suffer from the lightest of attentional blinks. More relevant, AVGs show an attenuated attentional blink^2,5,9,13^. These findings have been echoed in later training studies (e.g. Oei & Patterson, 2013).

As for adults, benefits of action video gaming experience have been observed in children and adolescents^3,14^. The underlying cause of attentional blink, however, is well-debated. The most widely-accepted account relates to a limitation of resources: a two-staged model which emphasises a significant role of target-discriminability^15^. According to this model, all stimuli enter an initial stage of perceptual and semantic processing^15^. In stage one, the stimulus activates its stored conceptual representation but this piece of information is volatile, susceptible to decay, and at risk of being overwritten by subsequent stimuli. In stage two, relevant target features initiate processing and trigger a temporary attentional response that leads to encoding of target into working memory^16^. Only those stimuli that have successfully undergone the capacity-limited stage two would be safe from overwriting and available for report^16^. In the case of an RSVP stream where the first target (T1) is temporally adjacent to the second (T2), they will be processed together because such short time difference between the targets allows T2 to enter the same temporal window as the first^16^.

A different, but related line of work considers the relationship between attention and spatial selectivity. Navon proposed that perceptual processing occurs in a temporally-organized manner, starting with global (structural) processing before fine-grained analyses^17^. Initial demonstrations^17^ revealed that normal observers responded quicker towards global information than to local information, and that incongruent local information did not affect response time of global level identification (although incongruous global information significantly affected local processing). That is, participants appear to have a particular sensitivity to global information – a preference that has sometimes been referred to as global precedence or bias in the literature^17^. Navon suggested that local features can still be processed but not without a deliberate effort^17^, and in the event of parallel processing, global processing would still finish earlier than local processing^18^. Other studies have also shown that global elements of attended and non-attended objects are harder to ignore as compared to local elements^19,20^. Of immediate relevance, research using hierarchically-structured stimuli has shown that individuals often have varying degrees of perceptual preferences, and that those with a preference towards global information, also demonstrate less attentional blink^21,22^. When placed into the context of visuo-attentional advantages in video-game experts demonstrated independently by a number of groups, these data drive an intriguing question: Might attentional advantages in video-game experts be related to greater global precedence as compared to non-experts?

Here, our question is two-fold: We ask if the attentional advantage for action video-gamers versus non-gamers, as demonstrated by a reduced attentional blink, holds both when attention is selectively directed at global or local information. We introduce locally and globally congruent or incongruent stimulus configurations while preserving the classical RSVP blink paradigm. Using the same paradigm, we also probe the possibility that attentional advantages, if any, could be simply explained by differing perceptual preferences (i.e., differing global precedences – stronger preference for global information in one group). We predicted that to the degree with which video-game experts show a reduced attentional blink, they may show a correspondingly greater global precedence. In two experiments, we tested both action video gamers and non-video gamers in a modified attentional blink task using Navon-type figures. We tested for differences in visuo-attentional capacities between the two groups both when asked to make global stimulus judgments (Experiment 1) and local judgments (Experiment 2) under conditions of both coherent and conflicting stimulus information.

## Methods

### Participants

A total of 119 participants were recruited through advertisements posted on university notice boards, explicitly requesting self-identified video-game players (at least 4 hours per week of game-play) and non-video-game players. Sixty-two of these participants participated in Experiment 1 (globally-oriented AB task), and the rest participated in Experiment 2 (locally-oriented AB task). In Experiment 1, observers ranged in age from 20 to 30 years old (*M* = 22.36, *SD* = 2.98), and were sorted into action video-gamers (AVGs) or non-video gamers (NVGs) based on their answers to a preliminary survey of their video gaming habits and expertise (regardless of their self-identified status, see below). Four participants were excluded from the study because although they self-identified to be action video-gamers, they in fact could not be classified clearly into either category based on their responses and our criteria. Among the remaining 58 participants, twenty-nine of these observers were categorised as AVGs (AVG: 18 males, 11 females; NVG: 11 males, 18 females). In Experiment 2, we tested fifty-five new observers after excluding two observers who could not be classified (AVG: 18 males, 10 females; NVG: 12 males, 15 females), who ranged in age from 18 to 35 years old (*M* = 22.2, *SD* = 3.03).

Our categorisation criteria followed Green and Bavelier’s landmark study^5^. Participants who engaged in action, action-adventure or shooter video games for at least four hours per week for the previous six months prior to the experiment were considered as AVGs. Participants who reported to have not continuously played for the six months immediately prior to the experiment, but have been involved extensively with action-type games in the past, were excluded. Participants reported playing action video games or action-adventure games such as League of Legends, Assassin’s Creed, Uncharted: Jake’s Fortune, Call of Duty and Jubeat. They also rated their level of expertise in relation to these, self-identifying as advanced but also occasionally as intermediate and master-level players.

The influx of new video games in the past decade and consequently, changes in visuo-spatial skills required by games on the consumer market allude to the need for new classifications to be used to fully understand the current gaming landscape. Under a revised sub-categorisation scheme^23^, action video games can be further broken down into first (FPS)/third (TPS) person-shooter, sports, real-time strategy (RTS), action-role-playing-(RPG), and action-adventure types, which still share common characteristics in terms of real-time combat (immediate visual feedback) and requirements of rapid responses. In this study, we considered action videogames to include first (FPS)/third (TPS) person-shooter, sports, action-role-playing (RPG), and action-adventure subtypes, which use the same action combat mechanics and have similar rapid response requirements. Traditionally, action video games included games such as Call of Duty, Halo, and Unchartered. Several newer titles, however, also fit action video-game criterion:

One such game is Jubeat – a title that is usually classified for the consumer as a music game. However, this game requires vigilant monitoring, fast motions, and simultaneous tracking of multiple targets, which well fit the criteria for action games, as adopted by previous action video game-related studies^6^. In Jubeat, the individual is required to tap multiple small grids on a large screen when prompted. These prompts appear in quick succession, in the periphery and/ or at the centre, and can be very distant from one another especially at high difficulty levels. Execution of this game always requires the action of at least two fingers on each hand. These properties render the requirements for this game akin to those for more traditional action video games. Finally, classification for another recent, and very popular game, League of Legends may appear ambiguous; in fact, this game belongs to an action-RTS or multi-player online battle arena (MOBA) game genre which taps on the cognitive systems as traditional action games^23^. Hence, it was also included here as fulfilling action-video-game criterion.

NVGs were considered as those who had zero or less than an hour of gaming for the past six months. As video games are an integral part of daily life, participants who played video games without fast motions, vigilant monitoring or any other aspect typical of action video-games still qualified as an NVG. Studies have shown that not all video games enhance visual attention. For example, strategic games such as Rise of Nations, slow-pace sport games including Harry Potter: Quidditch World Cup, role-playing games or the famous Tetris which belongs to simple visuo-motor game genre were not found to be beneficial to visuomotor skills and visual attention^3^. Thus, participants who have sporadically engaged in the above gaming genres were still categorised as NVGs in the present study.

With groups classified according to the above criteria, we confirmed that AVGs spent significantly more hours per week engaging in video game playing (*M* = 12.26 hours, *SE* = 1.42) than NVGs (*M* = 0.59 hours, *SE* = 0.23) [*t*(111) = −8.06, *p* < 0.001]. Most importantly, AVGs spent significantly more hours per week playing *action-type* video-games (*M* = 10.58 hours, *SE* = 1.20) than NVGs [*t*(111) = −8.73, *p* < 0.001]. There was no difference between the ages of the two groups [*t*(111) = 0.48, *p* = 0.633], nor differences in terms of years of education since primary one (AVG: [*t*(111) = 0.63, *p* = 0.53].

All participants provided written informed consent in line with procedures approved by the Research Ethics Board, The University of Hong Kong, and all methods conformed with the relevant guidelines and regulations. All participants had normal or corrected-to-normal vision as screened with the Snellen linear acuity chart.

### Stimuli and Apparatus

**Figure 1:**
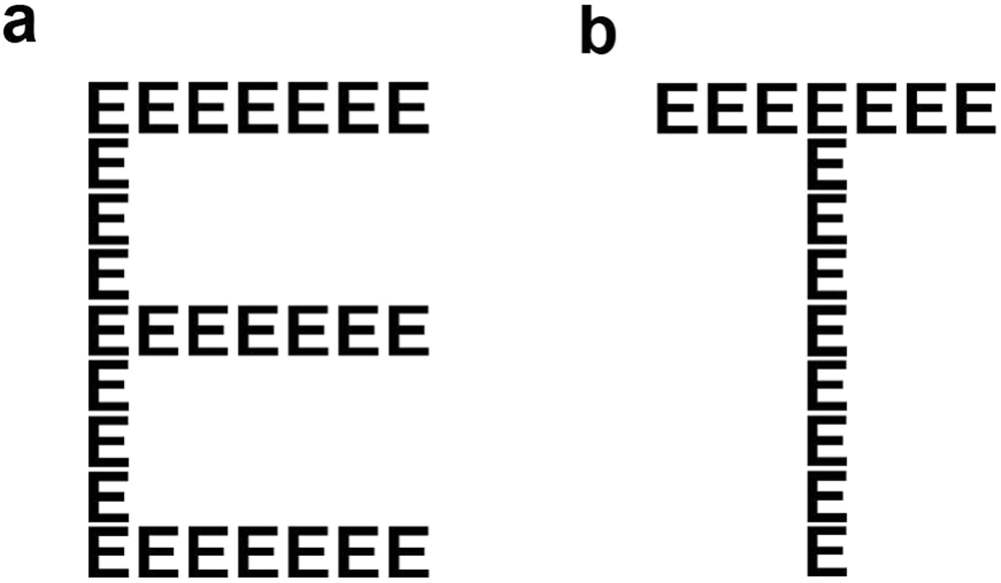
Sample stimuli (Navon figures) used in the modified attentional-blink task. Stimuli were composed of local and global elements, the identity of which could be congruent (**a**) or incongruent (**b**).

Stimuli were Navon-style figures composed of smaller letters arranged in a configuration that corresponded to a different, or same global letter as its local constituents (Fig. 1). These figures were produced in a non-serif style using select letters, E, F, H, L, N, S, T, U, Y, Z and X. The global configuration of the letter subtended visual angles of 6.1**°** × 4.2°, with its local elements each subtending 0.6° × 0.6°. Figures were either congruent (smaller and large letters are the same), or incongruent (large letter constructed of different smaller letters).

For all tasks, stimuli were presented in black (0.22 cd/m^2^), except for those designated “Target 1”, which were highlighted in white (135.72 cd/m^2^, see below) and centered against a mid-grey background (48.81 cd/m^2^). The number of local elements comprising each global figure, as well as the spacing between each local element was equivalent across all figures. Stimuli were generated and presented in Matlab^24^ using extensions from Psychtoolbox^25,26^. Stimuli were presented on a 24-inch Dell monitor with a refresh rate of 60 Hz. The experiments were conducted in an undisturbed, dimly lit room.

### General Procedures

Observers participated in either Experiment 1 (global task) or Experiment 2 (local task). The full experiment lasted approximately 45 minutes.

#### Experiment 1 (Global task)

In this variant of the attentional blink task, participants were asked to judge the global identity of the figures only. Each trial started with the presentation of a central fixation cross (0.5 × 0.5 deg) of two seconds followed by stimuli shown for 15 ms each, with each separated by a 85 ms interstimulus interval. The letter stream consisted of nine letters, among which T1 was always situated at the third temporal position. Since the temporal position of T2 relative to T1 determines the attentional blink, we elected to fix the position of T1, and vary the temporal position of T2. Introducing an additional manipulation for the temporal position of T1 would require a full counterbalancing of trials that takes into account that factor, together with that for T2 position (variable) as well as congruency (congruent/incongruent) – inflating trial requirements (i.e., experimental completion time) unnecessarily. T2, in the form of letter ‘X’, had a 50% chance of appearing after the presentation of T1. T2 was not presented immediately after T1 (i.e., at lag 1), as previous studies have reported a robust lag 1 sparing effect. T2 could instead appear at lag 2 (the fifth temporal position), lag 3 (the sixth temporal position) and lag 4 (the seventh temporal position). Note that as we were interested in testing the relative magnitude of attentional blink between the two groups, and not the absolute attentional blink and its recovery period, we elected to use these three lags that are typically encapsulated within the window expected to elicit an attentional blink, in order to maximise our sensitivity to any group-related differences^2,5,16,27^. Each stream terminated with a prompt asking participants to respond with the identity of T1 and subsequently, the presence of ‘X’ (yes/no) in the letter stream. Participants were given a maximum of three seconds to input their response after each prompt.

As the Navon figures could either be congruent (local and global identity identical) or incongruent (local identity differs from global identity), four figure conditions were presented within a block of trials: (1) T1 and T2 both congruent; (2) T1 and T2 both incongruent; (3) T1 congruent but T2 absent; (4) T1 incongruent but T2 absent. Participants completed a single run, where each condition was repeated 30 times, yielding to a total of 120 trials. Trial order was randomized and participants were allowed to rest briefly after every 40 trials. The entire task lasted 35 minutes. Prior to beginning the experiment proper, participants were given ten practice trials in which targets were shown at 100 ms to familiarize themselves with the task.

#### Experiment 2 (Local task)

Stimuli and procedures were identical to those of Experiment 1 except that new participants were asked to judge the local elements of the figures instead.

### Data Availability

The datasets generated during and/or analysed during the current study are available from the corresponding author on reasonable request.

## Results

### Global task

#### T1 identification accuracies between AVGs and *NVGs*

We first compared accuracies for T1 identification between the two groups by means of a 2 (congruency: congruent and incongruent) × 2 (group: AVGs and NVGs) mixed ANOVA. This initial analysis of T1 identification performance is critical to establish that the attentional blink is measurable (i.e., the attentional blink, by definition, requires the initial target to be detected and/or identified). The analysis indicated a marginally significant effect of group, with AVGs showing a subtle advantage for detecting T1 [*F*(1, 56) = 3.76, *p* = 0.058]. There was no significant main effect of congruency [*F*(1, 56) = 3.47, *p* = 0.068] nor a significant interaction between congruency and group [*F*(1, 56) = 3.16, *p* = 0.081].

#### T2 detection: only trials where T1 was correctly identified (the attentional blink)

**Figure 2:**
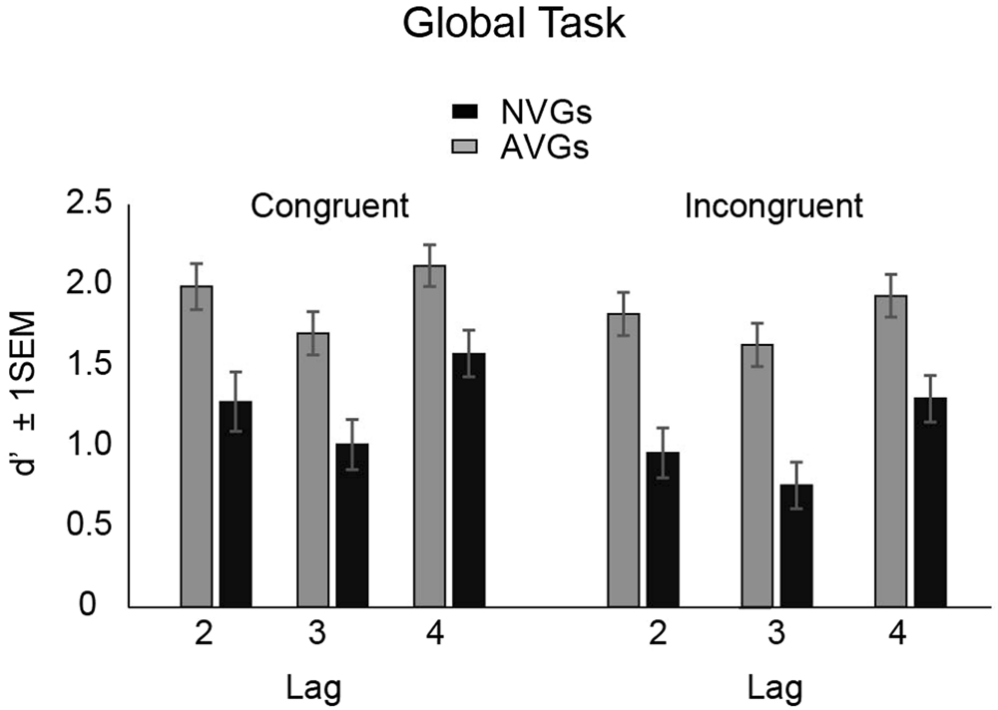
Target 2 detection performance for the *global task*, expressed in terms of d-prime sensitivities for both video-game and non-video-game experts. Note that Target 2 detection sensitivities are traditionally considered as the main measure of the ‘attentional blink’. These were computed for trials where T1 was correctly identified. Error bars represent +/− 1 SEM.

Next, we analysed T2 detection performance (dprime sensitivities), including only trials where T1 was correctly identified (Fig. 2). A 2 (group) × 2 (congruency) × 3 (lag) mixed ANOVA revealed significant a main effect of lag [*F*(2, 112) = 23.36, *p* < 0.001] and congruency [*F*(1, 56) = 8.35, *p* = 0.005]. There was also a significant main effect of group, reflecting higher sensitivities for AVGs as compared to NVGs [*F*(1, 56) = 20.02, *p* < 0.001]. Follow-up Bonferonni-corrected t tests again indicated that performance at lag 3 [*t*(56) = −4.20, *p* < 0.001] was significantly worse than that at lags 2 [*t*(56) = −3.79, *p* < 0.001] and 4 [*t*(56) = −3.27, *p* = 0.002]. Other interactions were not significant.

**Figure 3:**
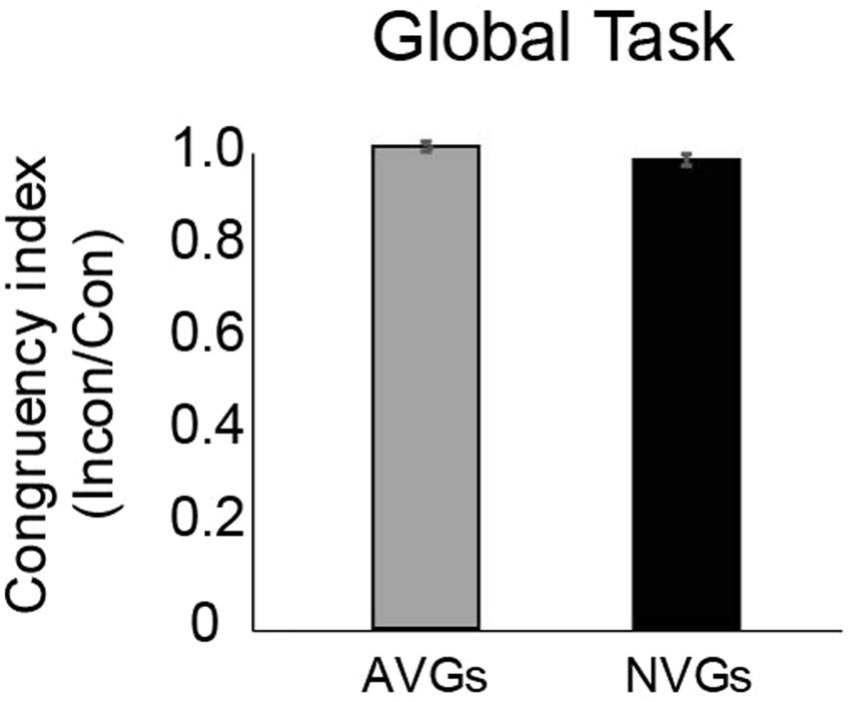
Target 1 identification performance for the *global task*, presented for both experts and non-experts in terms of a congruency index, computed as performance accuracy for incongruent trials divided by performance accuracy for congruent trials. A perceptual precedence for global information should be reflected in indices around 1. Notably, both groups are comparably affected by stimulus congruency. Error bars represent +/− 1 SEM.

The lack of a congruency and group interaction mirrors the lack of interaction observed for T1 identification accuracies, and, taken together, suggests that the overall attentional advantage for action video gamers cannot be attributable to differences in perceptual preferences; that is a larger global precedence (or preference) in AVGs versus non-experts^21,22^ despite having overall better global recognition ability (i.e., overall higher T1 identification performance). To unpack this idea, consider that an overall large preference for attending to global information would manifest itself in comparable performance between congruent and incongruent conditions (in the context of this global task). We can quantify this by computing congruency indices - obtained by dividing T1 accuracies of incongruent trials by those of congruent trials. Computed in this manner, an index close to 1 would indicate a lack of interference of conflicting local information, and provide a measure of global precedence (or preference) [AVG: *M* = 1.00, *SE* = 0.10; NVG: *M* = 0.97, *SE* = 0.01]. A comparison of indices for two groups would provide a measure of the relative interference of incongruent local elements between the two groups. Analyses of these indices indicated that while the indices of both groups were no different from 1 [Bonferonni-corrected *t* tests versus 1;* t*(28) = 0.10, *p* = 0.91 for AVGs; *t*(28) = −2.3, *p* = 0.03 for NVGs], there was no difference between the indices of the two groups [*t*(56) = −1.82, *p* = 0.07] (Fig. 3).

### Local task

#### T1 identification accuracies between AVGs and NVGs

As for Experiment 1, we first analysed the local task data in terms of T1 identification performance, quantified in terms of proportion of accurate responses. A 2 (congruency: congruent and incongruent) × 2 (group: AVGs and NVGs) mixed ANOVA indicated a main effect of congruency [*F*(1, 53) = 39.71, *p* < 0.001], reflecting the fact that T1 identification performance was better for congruent than incongruent trials. Notably, there was no significant main effect of group; that is, AVGs did not perform any better than NVGs in terms of local T1 identification [*F*(1, 53) = 0.89, *p* = 0.35]. There was also no significant interaction between stimulus congruency and group [*F*(1, 53) = 0.27, *p* = 0.61].

#### T2 detection: only trials where T1 was correctly identified (the attentional blink)

**Figure 4:**
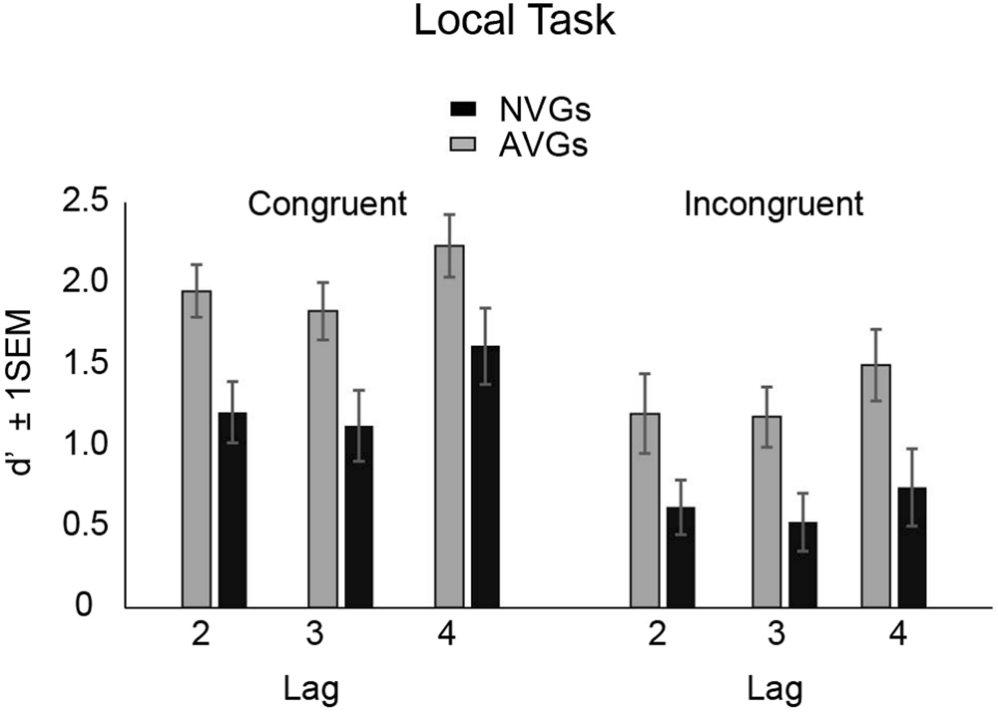
Target 2 detection performance for the *local task*, expressed in terms of d-prime sensitivities for both video-game and non-video-game experts. These were computed for trials where T1 was correctly identified. Error bars represent +/− 1 SEM.

Next, we considered T2 detection performance only for trials where T1 was correctly identified (Fig. 4). The data were entered in to a 2 (group) × 2 (congruency) × 3 (lag) mixed ANOVA. This analysis indicated a significant main effect of group, [*F*(1, 53) = 8.53, *p* = 0.005] reflecting the fact that T2 detection was generally better for AVGs versus NVGs. There were also significant main effects of lag [*F*(2, 106) = 9.19, *p* < 0.001] and congruency [*F*(1, 53) = 38.55, *p* < 0.001]. Neither the lag by congruency interaction nor congruency by group interaction was significant. Further analysis of the main effect of lag indicated that performance at lag 3 [*t*(53) = −3.19, *p* = 0.002] was significantly lower than that at lags 2 [*t*(53) = −2.88, *p* = 0.006] and 4 [*t*(53) = −2.57, *p* = 0.013]. Other interactions were not significant.

**Figure 5:**
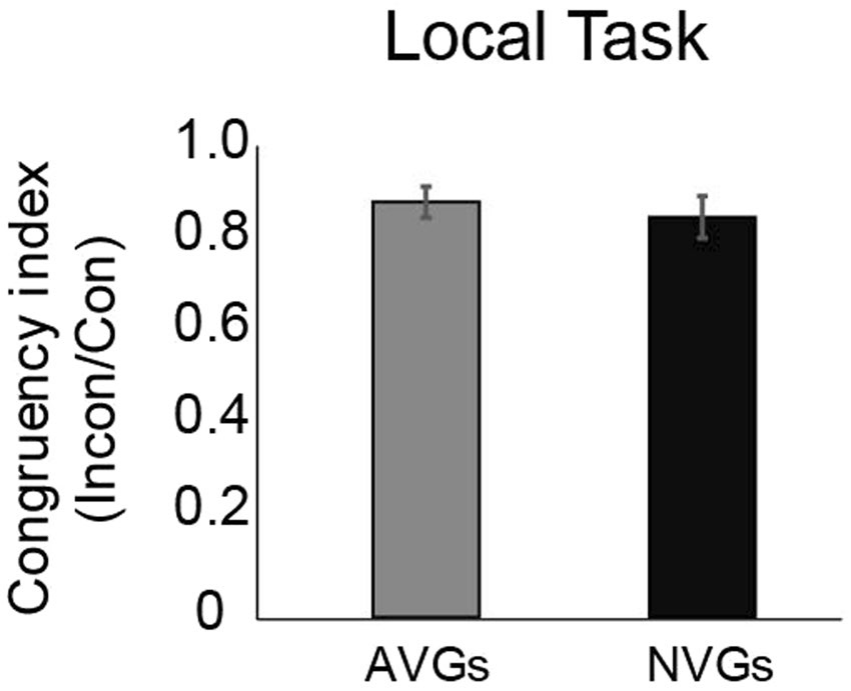
Target 1 identification performance for the *local task*, presented for both experts and non-experts in terms of a congruency index, computed as performance accuracy for incongruent trials divided by performance accuracy for congruent trials. A perceptual precedence for global information for this particular task should be reflected in indices of less than 1. Notably, both groups are again comparably affected by stimulus congruency. Error bars represent +/− 1 SEM.

As for the global task, there was no interaction between group and congruency. As for Experiment 1, we illustrate the lack of interaction between group and congruency on T1 performance, by computing congruency indices, obtained by dividing performance on incongruent trials by that on congruent trials (as described earlier) (Fig. 5). Here, any global precedence (preference) would manifest itself in indices of less than 1, as local judgments would be largely interfered with incongruent global information [AVG: *M* = 0.84, *SE* = 0.03; NVG: *M* = 0.81, *SE* = 0.04]. The indices computed as such were significantly less than 1 for both the AVG [Bonferonni-corrected *t* tests versus 1; *t*(27) = −4.9, *p* < 0.001] and NVG [*t*(26) = −4.36, *p* < 0.001] groups, but not significantly different between the two groups [*t*(53) = −0.59, *p* = 0.56], again indicating the degree of global precedence is comparable between the two groups.

In sum, AVGs showed overall better performance than non AVGs in terms of T1 identification for the global, but not local task. Considering the attentional blink (T2 target detection), AVGs showed attenuated blinks (i.e., greater sensitivity for detecting T2) for both global and local tasks. Importantly, however, this advantage was uniform across stimulus congruency in both the global and local tasks, and cannot be simply attributable to enhanced ability to detect/identify T1 in AVGs as any potential group advantages were removed already by considering only trials where T1 were correctly identified. These data suggest that the smaller ‘blink’ observed for AVGs is not attributable to overall better global percepts, nor attributable to differing perceptual tendencies - global precedence effects (see earlier discussion on the expected manifestation of such differences in global precedence in terms of our congruency effects).

## Discussion

In light of independent lines of work showing advantages for action video gamers for visual attention^1,2,5–9^, we probed the interaction between visuospatial attentional requirements and video-gaming expertise. We used a modified attentional blink paradigm involving Navon-type stimulus figures that were congruent or incongruent and asked observers to direct their attention globally (Experiment 1) or locally (Experiment 2). This setup allowed us to probe the extent to which expertise-related advantages in visual attention could be attributable to global precedence. Action video-gamers showed a significantly shallower attentional blink, quantified by higher T2 detection sensitivities. These findings are congruent with attentional advantages shown previously for video game experts^2,5^. Still, as noted earlier, despite the large body of literature that align with our findings, other studies have failed to find clear attentional advantages in video game experts^10,11^. Several factors may contribute to the apparent discrepancy in the literature.

The first factor relates to video-gaming habits. The vast majority of habitual gamers in our study engaged purely in action video-games whereas the gaming habits of participants in other studies may centre around strategy-type games – games that may not require the same degree of visuo-motor precision nor attentional requirements.

Likewise, performance seems to be highly dependent on the type of attentional blink task. In a recent study, the probe task employed by Green and Bavelier was replaced by a categorical test^5^, in which participants were required to identify numbers from letters^27^. The task was evidently easier because of increased target-distractor discriminability and longer target exposure time. Probe tasks require recalibration of the input filter, which is not required in a categorical and feature tasks^28^. Furthermore, varying lags at which T2 would appear may mask any attentional blink effects^10^.

One last factor that has gained traction in the recent literature in terms of potential influences on empirical work is broader, but perhaps still relevant, and relates to the method of recruitment. In the present study, advertisements included explicit requests for video-gamers and non-video-gamers; hence participants were initially self-identified. Upon arriving in the laboratory, they were then required to complete a formal screening survey in order for us to perform formal classification. We deem overt recruitment as having been important in order to ensure we did not consistently recruit participants that required disqualification, which would likely be the case under covert circumstances since participants with some casual or former intensive gaming history would be excluded from either group. In order to minimise any potential expectation effects, participants were not provided any information as to the purpose or hypotheses of the study, and were also instructed that the screening form constitutes a part of the standard battery of tests for visual screening (i.e. it was conducted together with the linear acuity test). Literature that has looked directly at the effects of knowledge of a study’s purpose has not shown clear trends^29, 30^. Perhaps more importantly, attentional blink is concerned with attentional capacities which appear to be difficult to alter at will. Furthermore, previous action video game studies that have used covert recruitment^31^ have produced findings very comparable to those that have used overt advertisement strategies^7,14,32^. Nonetheless, as both groups were recruited in the same manner, any overall enhancements or degradations of performance due to demand characteristics should be comparable for both groups.

In the present experiments, attentional blink was strongest for both groups at lag 3, recovering somewhat at lag 4 (Fig. 2). Thus, the attentional drop (and consequently, the recovery) is *delayed*. As we did not seek to test the temporal window needed to achieve full recovery (i.e., 100% correct-rate T2 detection), previous attentional blink work utilising Navon letter figures in RSVP streams have indicated that it takes a significant period for performance to recover^33,34^. In one study^33^, participants attained a T2 detection performance of 60% accuracy at 1 second post-T1 offset, and required 1.52 seconds (post-T1 offset) to reach 80% correct identification of T2.

The pattern of results obtained when observers were instead asked to direct their attention to the local elements (Experiment 2) were largely similar to those obtained in the global attention task. One notable exception to this was that while AVGs showed a clear advantage for identifying global letter identity, this advantage disappeared when the two groups were instead asked to judge the local elements. As for the global task however, both groups showed significant global precedence (as reflected by congruency indices, Figs 3,5). Perhaps more importantly, performance of both groups (in both tasks) was comparably affected by congruency, not only in terms of T1 identification performance but also in terms of T2 detection sensitivities, suggesting comparable levels of global precedence. We interpret these data to suggest that the extent to which action video gamers exhibit visuo-attentional benefits (i.e., smaller attentional blinks) is unlikely to be related to their degree of global precedence. Still, we caution that while our conclusions are based upon the lack of observable differences in terms of congruency effects between the two groups in the present data (both pertaining to T1 identification, and T2 attentional blink), they are limited in a sense that we are unable to quantify the same base relationship between global precedence and general attentional blink as presented by Dale and Arnell^21,22^. This is, at least in part, due to design-level differences. Dale and Arnell^21^ demonstrated that global precedence, as retrieved from performance (reaction times) on a global/local task involving Navon-type figures could predict attentional blink magnitude in a second task using standard (non-Navon-type) elements. To quantify global precedence, the authors first computed measures of *global interference* (the amount of interference from global items during local judgments), and *local interference* (the amount of interference from local items during global judgments). Global precedence was then computed as the difference between global interference and local interference.

Given our choice of design, we are unable to reproduce the same metric of global precedence as we opted for a between-subject assessment of the global and local tasks. Moreover, our measures of performance are based on identification accuracies (T1), rather than reaction time data, which would be uninformative here as the T1 response probe occurs after the offset of the entire RSVP stream. Still, we attempted to reconstruct a comparable analysis to that of Dale and Arnell^21,22^, using the present data. Specifically, we first computed comparable measures of global and local interference using the T1 identification data. Local interference was quantified by computing the difference between Target 1 accuracies in the incongruent versus congruent conditions in all trials during global judgments. A value greater than zero then, suggests a degree of global precedence. Correspondingly, global interference was quantified by computing the difference between Target 1 accuracies in incongruent versus congruent conditions during local judgments. A value less than zero in this case, suggests global precedence.

We then computed additional measures of local and global interference using the attentional blink data, by obtaining the difference between Target 2 detection sensitivities in the incongruent versus congruent conditions (given Target 1 was correctly identified). Finally, we entered these data into simple correlational analyses that revealed no evident relationship between either global or local interference with the attentional blink in either group (see Supplementary Figure S1 for details).

We speculate that additional methodological differences may also contribute to discrepancies with Dale and Arnell’s evaluations of the attentional blink (and consequently, its relationship to global precedence)^21,22^. Any of the differences in stimulus duration, interstimulus interval, percentage of trials which T2 was present, temporal position of T1 and RSVP stream length could have contributed to a difference in sensitivity for measuring both the attentional blink and global precedence within the stream.

Critically, video game experts again showed attenuated attentional blink as compared to non-experts in the current study. Across both experiments, both groups performed worst at lag 3, marking a sharp decline from lag 2 – the temporal position that usually elicits the largest blink in conventional attentional blink paradigms^12,16^. While the standard attentional blink delay and lag sparings have been explained in the context of Chun and Potter’s two-stage model^15^, we suggest that the Boost and Bounce model^35^ could perhaps better explain the ‘delayed drop’ observed here at lag 3. The model suggests that a ‘spread of sparing’ to lags that are temporally near T1 is possible because the attentional blink is caused by the subsequent fall from a strong attention boost required for recognising T1. According to this model, targets at lags 2 and 3 can still be processed and enter working memory provided that the upcoming stimulus and T1 both belong to the same stimulus class set^36,37^.

Similar to the difficulties previous video-game-related attentional studies have encountered^27,38,39^, we were unable to achieve a gender balance between our video-gaming and non-video-gaming participant groups. Complicating evaluations of the effect of such gender imbalance on our effects is the fact that the current literature suggests that males perform better than females in some (i.e., mental rotation), but not all tasks that involve spatial attention; males also tend to show more variability in their performances on these tasks^40^. In the context of video-game expertise, one study found gender differences in spatial attention among NVGs when tested with a UFOV paradigm^9^. Still, a previous study using an attentional blink paradigm reported negligible effects of gender^14^. In order to address potential concerns about gender effects lurking in our data, we plotted T2 detection sensitivities separately for males and females (Supplementary Figure S2). An initial examination of these figures revealed no discernible differences in patterns of effects between the two genders. We subsequently performed additional analyses on our attentional blink data, including gender as a factor. Results from these analyses suggest that expertise-related effects found in our current experiments are comparable for both gender groups, and for both tasks (See Supplementary Figure S2), echoing current literature which has not recorded clear gender differences both in visual processing at large and in visual attention^40–44^.

Finally, it is interesting to speculate on the mechanisms tapped into by our two tasks. Neuroimaging work has shown that global and local attention selectively activate differing areas in the temporal-parietal cortex, with attention to global figures activating the right lingual gyrus, and attention to local features activating the left inferior occipital cortex^45^. Moreover, psychophysical work has indicated that global attention displays shorter (more transient) blinks than the local attentional system^34^. This characteristic of the global system is intriguing as it has been argued that transient visual attention is driven largely by the magnocellular system^46^. M-cells have larger receptive fields, transient responses, and higher contrast sensitivity^47^. Therefore, the M-pathway is especially important when presented with fine temporal information^48^. Could the attenuated attentional blink observed for video game experts be reflective of an enhanced M-system?

### Summary

Using a modified attentional blink paradigm employing Navon-type hierarchical figures, we showed that congruent with early studies^2,5,9^, action video game experts exhibit an attenuated attentional blink relative to non-experts. This benefit holds both when attention was selectively directed towards global or local features. Critically, this apparent visuo-attentional advantage is independent of group differences in attaining general task demands (i.e., is present even when we equate for target identification ability). The attentional benefits also cannot be well explained by greater (or local) precedence perceptual tendencies^21,22^, as both groups were comparably affected by stimulus congruency. Instead, the observed advantages may be indicative of a fine-tuned selective attention system adept at resolving temporally-transient information, perhaps manifest in an enhanced Magnocellular system.

## Electronic supplementary material

Supplementary information
