# Supplementary material for: Attentional advantages in video-game experts are not related to perceptual tendencies
Source: Sci Rep. 2018 Apr 3;8:5528. doi: 10.1038/s41598-018-23819-z (PMC5882918; doi:10.1038/s41598-018-23819-z)
Supplement: Supplementary file 1 — Supplementary information [file 41598_2018_23819_MOESM1_ESM.docx]

Attentional advantages in video-game experts are not related to perceptual tendencies

Nicole H. L. Wong & Dorita H. F. Chang

Submitted to: *Scientific Reports*

**Supplementary Information**

Figure S1. Correlation between local and global interference and attentional blink for video game experts and non-experts.

Figure S2. Target 2 detection performance for the global and local tasks between males and females, independently for video game experts and non-experts.


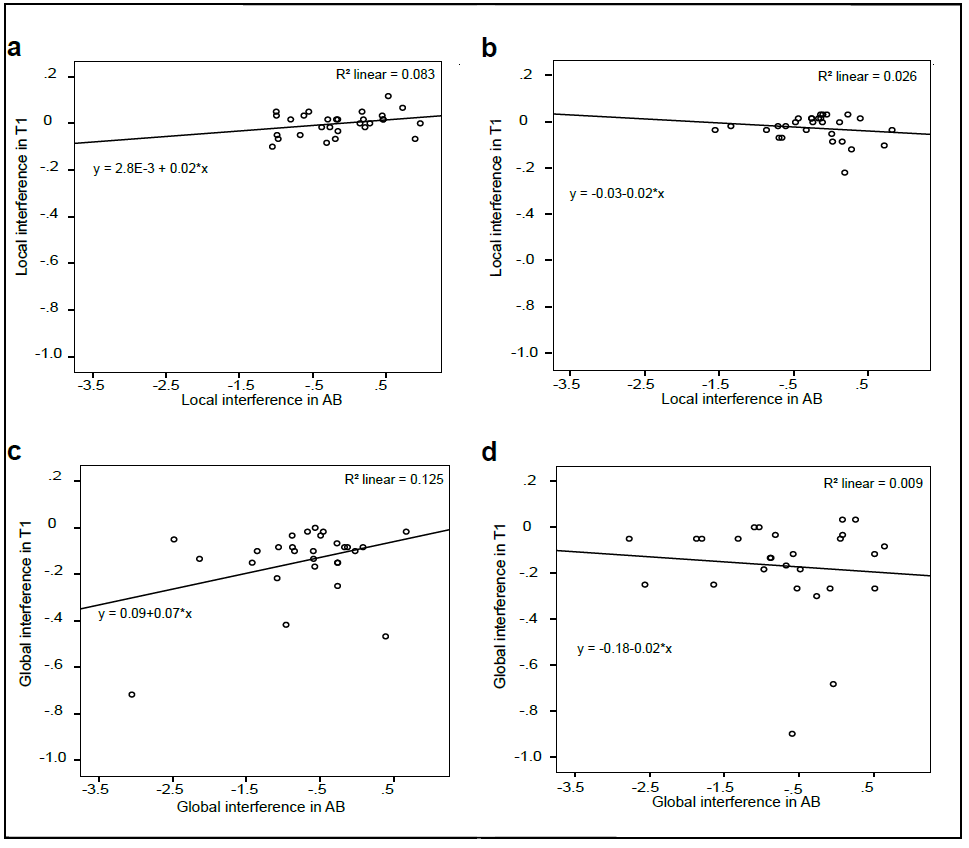
**Figure S1**

**Figure S1.** Correlation between the attentional blink, and measures of local or global interference presented independently for action video game experts and non-experts. *Local interference* was quantified by computing the difference between Target 1 accuracies in incongruent versus congruent conditions in all trials during global judgments. A value greater than zero then, suggests a degree of global precedence. Correspondingly, *global interference* was quantified by computing the difference between Target 1 accuracies in incongruent versus congruent conditions during local judgments. A value less than zero in this case, suggests global precedence.

For the computation of local and global interference in the attentional blink, we computed the difference between Target 2 detection sensitivities in the incongruent versus congruent conditions (given Target 1 was correctly identified). Results of these comparisons are presented for (a, b) AVGs in the global and local tasks, respectively, and (c, d) NVGs in the global and local tasks, respectively.

Correlational analyses neither indicated a significant relationship between local interference and attentional blink (AVG: *R^2^*= 0.08, *r* = 0.29, *p* = 0.13; NVG: *R^2^* = 0.03, *r* = -0.16, *p* = 0.40), nor a significant relationship between global interference and attentional blink for non-experts (*R^2^*= 0.009, *r* = -0.09, *p* = 0.64), and for action video game experts (*R^2^*= 0.013, *r* = -0.35, *p* = 0.07).


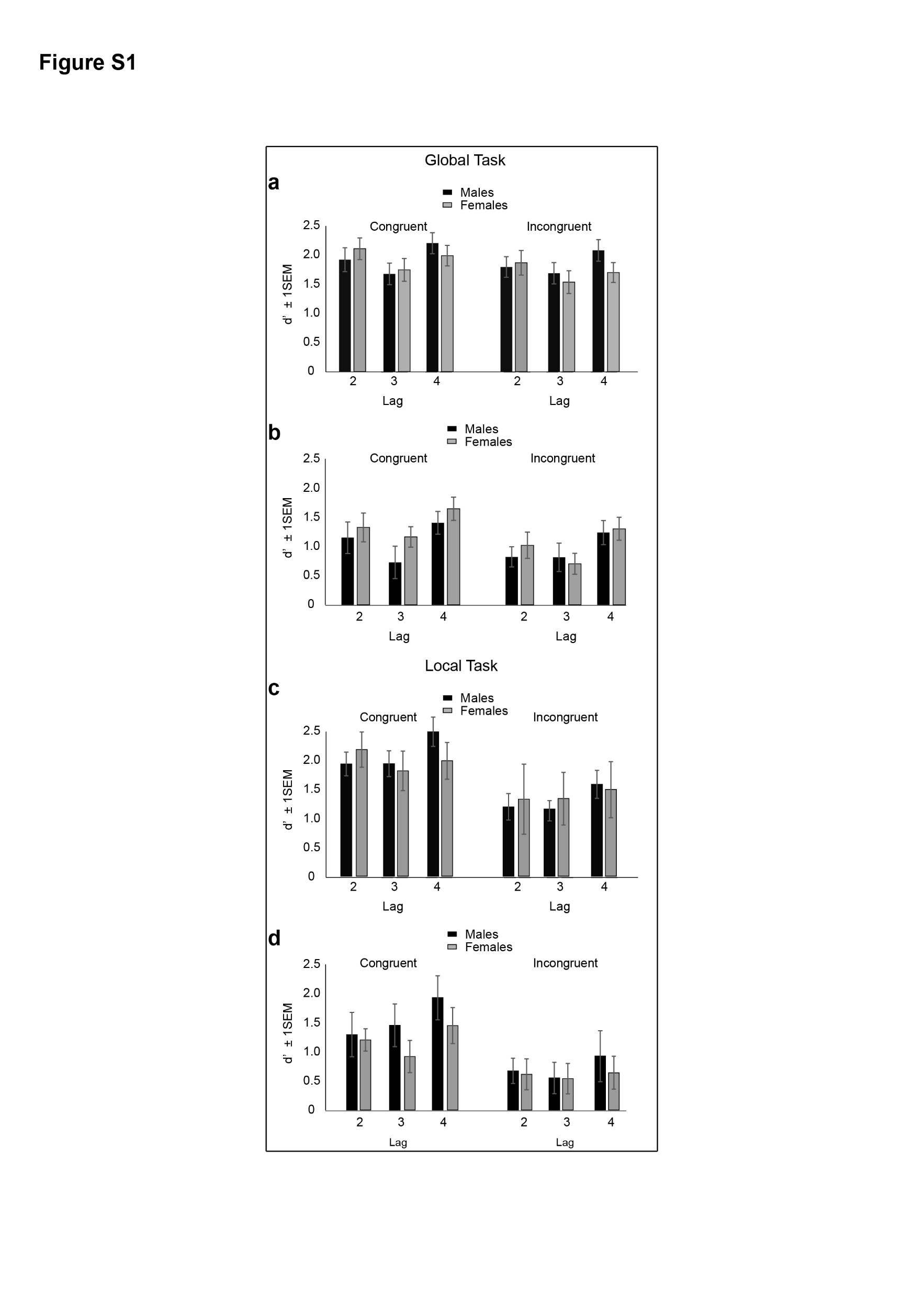
**Figure S2**

**Figure S2**. Target 2 detection performance (computed as d-prime sensitivities) presented separately for males and females, for the global and local tasks, and independently for action video game experts and non-experts. D-prime sensitivities are presented for (a, b) AVGs and NVGs respectively for the global task, (c, d) AVGs and NVGs respectively for the local task. Error bars represent +/- 1 SEM. An initial examination of these figures revealed no discernible differences in patterns of effects between the two genders.

For the global task, a 2 (congruency) x 3 (lag) x 2 (gender) mixed ANOVA analysis indicated no significant differences in T2 sensitivities between genders [*F*(1,56) = 0.46, *p* = 0.50], and no interactions involving gender [congruency x gender: *F*(1,56) = 2.37, *p* = 0.13; lag x gender: *F*(2,112) = 0.80, *p* = 0.451; congruency x lag x gender: *F*(2,112) = 0.99, *p* = 0.375].

A comparable analysis for the local task indicated again no significant difference in T2 sensitivities between the two genders [*F*(1,53) = 1.12, *p* = 0.294], and no interactions involving gender [congruency x gender: *F*(1,53) = 0.94, *p* = 0.338; lag x gender: *F*(2, 106) = 2.336, *p* = 0.099; congruency x lag x gender: *F*(2,106) = 1.11, *p* = 0.33].
